# How much does HIV self-testing cost in low and middle income countries? A systematic review of evidence from economic studies

**DOI:** 10.3389/fpubh.2023.1135425

**Published:** 2023-11-13

**Authors:** Brianna Empringham, Angela Karellis, Abdulhameed Kashkary, Olivia D’Silva, Sergio Carmona, Marta Fernandez Suarez, Angelina Addae, Nitika Pant Pai, Alice Anne Zwerling

**Affiliations:** ^1^School of Epidemiology and Public Health, University of Ottawa, Ottawa, ON, Canada; ^2^Children’s Hospital of Eastern Ontario, Ottawa, ON, Canada; ^3^Division of Clinical Epidemiology & Experimental Medicine, McGill University, Montreal, QC, Canada; ^4^Public Health Authority, Riyadh, Saudi Arabia; ^5^Foundation for Innovative New Diagnostics, Geneva, Switzerland

**Keywords:** cost effectiveness, health systems, HIV epidemiology, testing, systematic review

## Abstract

**Objectives:**

HIV self-testing (HIVST) has been proposed as an innovative strategy to diagnose human immunodeficiency virus (HIV). While HIVST offers the potential to broaden accessibility of early HIV diagnosis and treatment initiation, this testing strategy incurs additional cost and requires confirmatory testing and treatment. We have conducted the first systematic review to summarize the current economic literature for HIVST in low- and middle-income countries (LMICs).

**Design:**

A search strategy was developed including key terms for HIV, self-testing and cost-effectiveness and was conducted in Medline and Embase databases. Studies were included that reported costs per outcome and included both cost-effectiveness and cost-utility outcome measures. The search strategy identified publications up until August 15, 2023 were included. Abstract and full text screening was conducted and a standardized data abstraction form was used for included studies. Costs are reported in USD, 2020.

**Results:**

Our search strategy identified 536 total titles from the search strategy, which were screened down to 25 relevant studies that provided both cost and outcome data on HIVST. There was significant heterogeneity in the HIVST intervention, study population, costs and outcomes reported among included studies. Cost per person tested ranged from $1.09–155. Cost per case diagnosed ranged from $20–1,277. Cost-utility estimates ranged from cost-saving to $1846 per DALY averted. Higher cost-effectiveness estimates were associated with more expensive testing algorithms with increased support for linkage to care and post-test counseling.

**Conclusion:**

All studies considered HIVST cost-effective although major drivers were identified included underlying HIV prevalence, testing cost and linkage to care. HIVST is likely to be cost-effective in a LMIC context, however policy makers should be aware of the drivers of cost-effectiveness when implementing HIVST programs as these underlying factors can impact the overall cost-effectiveness of HIVST.

## 1. Introduction

HIV self-testing (HIVST) has been proposed as an innovative strategy to diagnose human immunodeficiency virus (HIV) (1). HIVST allows an individual to collect their own specimen (via sputum or blood) and conduct/interpret the HIV test independently or with support from a health care worker, HIVST can be performed within the individual’s home or in a healthcare facility. Individuals with positive self-tests would then follow up with confirmatory testing (linkage to care) and post-test counseling (2).

Previous literature has strongly suggested that HIVST is preferred by clients and has increased rates of uptake compared to conventional testing (3–7). This has been shown among the general population, but also among conventionally hard to reach groups such as sex workers and truck drivers (8, 9). One potential concern raised around HIVST is ensuring access to confirmatory testing and high rates of linkage to care (1, 10, 11). To address this potential limitation, HIVST is increasingly delivered with community or digital based supports which can help increase testing uptake, interpret results and support linkage to care (12). In December 2020, the UNAIDS set out a goal to diagnose 95% of HIV positive individuals, start ART for 95% of diagnosed individuals and achieve viral suppression for 95% of individuals on ART (13). As countries move towards achieving the 95–95-95 targets, HIVST offers a testing method that is acceptable to communities and empowers individuals to engage in their own health, rather than relying on a facility-based testing approach.

While HIVST offers the potential to broaden accessibility of early HIV diagnosis and treatment initiation, implementing a novel testing strategy incurs additional cost and requires individuals with positive self-tests to follow up with confirmatory testing and treatment. While previous studies have suggested that HIVST can be cost effective (14, 15), understanding of this growing field is still limited and this is the first systematic review to summarize the economic literature around HIVST. The objective of this review was to describe the current literature for the cost-effectiveness of HIVST within low- and middle-income countries (LMIC). Research suggests that the rates of follow up to confirmatory testing are highly variable based on the implementation approach, therefore our results have been stratified by both study type and distribution strategy (2, 10–12).

## 2. Materials and methods

A comprehensive literature search was performed with the assistance of a medical librarian to identify current studies of HIVST combined with an economic filter in low- and middle-income countries.

**Figure 1:**
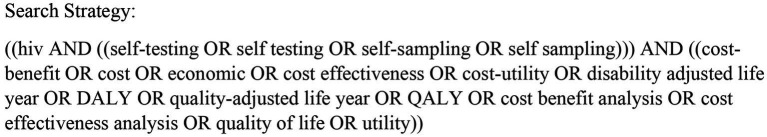
Outlined above is the search strategy that was used to conduct the systematic review in both Medline/Pubmed and Embase databases.

The search was conducted in two databases: Medline/PubMed and Embase and included all publications from inception through August 15, 2023. The search was initially conducted in October 2021 and then updated in August 2023. Given that HIVST is a relatively new intervention, to capture further eligible studies, bibliographies were searched and reviewed. Search terms used included “HIV,” “self-testing,” “self-sampling” and economic terms such as “cost-effectiveness,” “cost-utility” and “quality of life” (Figure 1).

The inclusion criteria for extraction were as follows: an economic evaluation study design of HIVST intervention (initiation or continuation), conducted in low or middle income countries as defined by World Bank, peer-reviewed, and published in English (16). This included studies that assessed the cost-effectiveness, cost-utility, cost–benefit and cost-minimization of a HIVST intervention. These included but were not restricted to the following outcomes: (i) cost per quality-adjusted life years; (ii) cost per HIV self-test; (iii) cost per diagnosis of HIV. Studies that created modeled cost effectiveness and outsourced model inputs from previous literature were included. Studies were excluded if they were not conducted in low- or middle-income country as defined by the World Bank, were not original, peer-reviewed research articles (reviews, monographs, and conference abstracts) or did not report costs associated with HIVST.

Study selection and presentation followed PRISMA (Preferred Reporting Items for Systematic reviews and Meta-Analyses) guidelines (17). Two independent reviewers (AnK, AbK) selected eligible publications initially based on titles and abstracts. Articles that met the inclusion criteria were retained for data extraction. At any stage in the review process, any disagreements were resolved by a third-party reviewer. Full text reviews were completed, and data was extracted from eligible studies (BE, AnK, and AbK) utilizing a standardized data abstraction form. The extracted study data was summarized in narrative format and stratified by study type and HIVST distribution strategy. Grouping studies by distribution strategy allowed us to compare results among similar HIVST interventions. Results are also reported stratified by outcome measure. Given the underlying heterogeneity of included costs and outcome measures of the included studies, a meta-analysis was not conducted. The methodological quality of HIV self testing economic evaluations was assessed using the 28-item Consolidated Health Economic Evaluation Reporting Standards (CHEERS) checklist (1). Each item was scored “Yes” if it met the quality criterion, or “No” if it did not meet the criterion. A numeric score was calculated for each study. “Yes” responses were weighed against the total number of criteria for percentage. Studies were assessed into three categories: high (above 75%), average quality (50–75%) and low quality (less than 50%).

All costs are reported in USD 2020. Costs were converted back into their original currency, adjusted for inflation (18) and converted to USD 2020.

## 3. Results

### 3.1. Study selection

**Figure 2:**
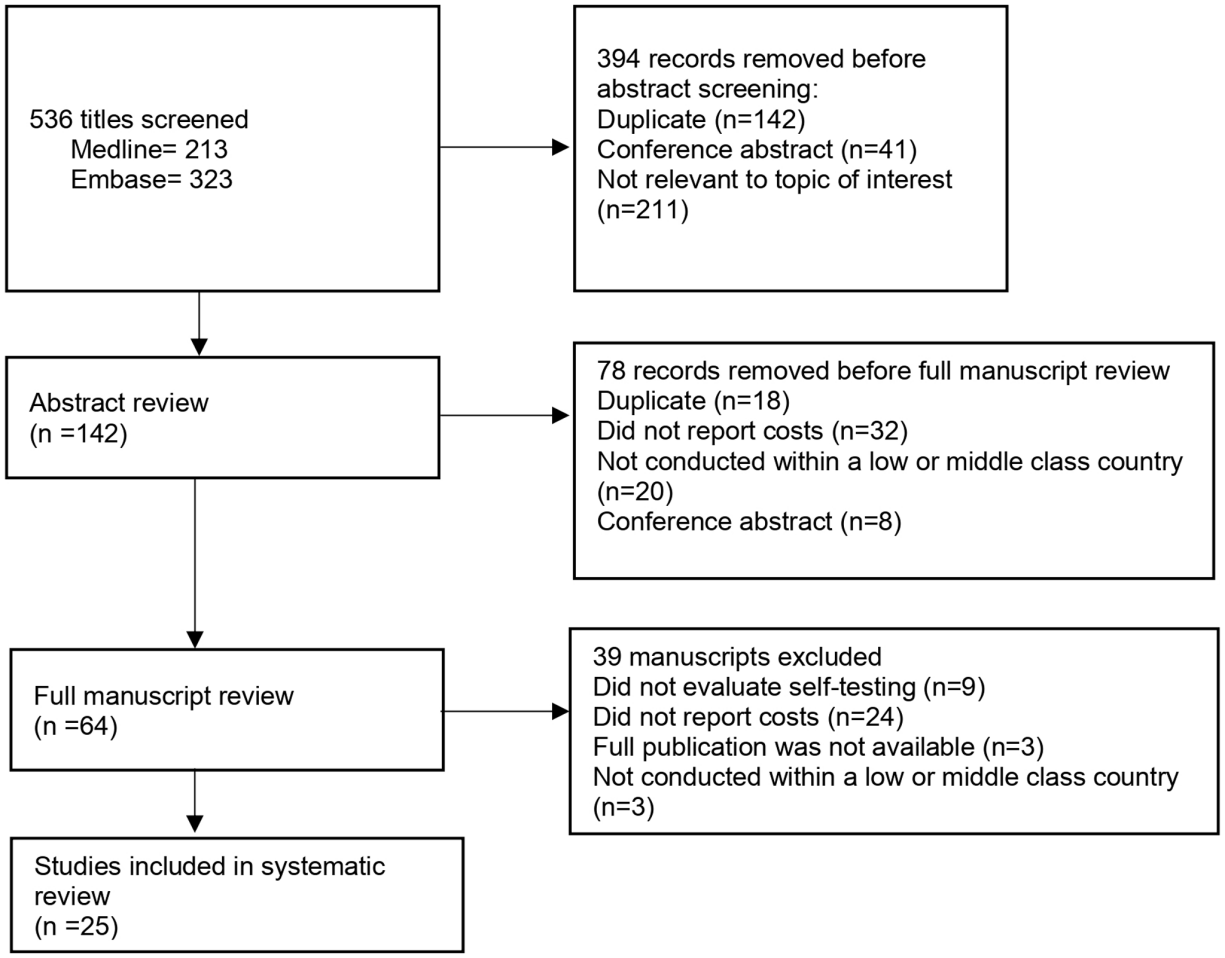
PRISMA flow diagram of systematic review.

The search strategy yielded 536 studies, of which 142 underwent full abstract review, a set of 64 full text articles were reviewed (Figure 2). Finally, 25 articles were included in this report. The search strategy and data extraction was conducted over a course of 2 months.

Of these 25 eligible studies, 17 were empiric in nature meaning that an actual intervention was conducted and costing data per outcome measure were reported. The other eight publications were modeling studies. A summary of the cost-estimate findings, stratified by outcomes measure is included in Table 1. The study characteristics and individual economic results are reported in Table 2.

**Table 1:** Study details.

Study, year	Type of study	Location	Study population	Self screen	Reference strategy	Analysis horizon	Perspective	Outcome assessed	Outcome result
Amstutz, 2021	Empirical	Lesotho	Individuals (>12 years) with unknown HIV status	Self-tests were distributed to individuals who refused home based HIV testing	Home based HIV testing (provided by a health care worker)	5 months	Health system	Cost per person tested	$16
								Cost per person diagnosed	$923
								ICER per person tested	$5
								ICER per person diagnosed	$142
Borgstede, 2023	Empirical	Southwestern Kenya	Pregnant women and their partners	HIVST (home-based) provided by mail along with counseling on post-test services	Home-based counseling FBT	1 month	Health system + patient-incurred	Cost per test	30.49–56.59
Bulterys, 2020	Empirical	Uganda	Pregnant women living with HIV and their male partners	Self-test conducted at home followed by clinic-based confirmatory test, counseling and linkage to treatment	Facility-based testing	5 years	Health system	Cost per female self-tested	Counseling provided individually: $12–18 Counseling provided in group sessions: $5
								Cost per male self-tested	$16–23
Cambiano, 2015	Modeling	Zimbabwe	General public (18–65 years)	Self-test (alone) followed by confirmatory test	Facility-based testing	20 years	Health system	ICER per DALY averted	$27,165 saved per DALY averted
Cambiano, 2019	Modeling	Sub-Saharan Africa	Women having transactional sex Young people (15–24 years) Adult men, (25–49 years)	Community-based self-test followed by confirmatory testing and counseling	Facility-based testing	50 years	Health system	ICER per DALY averted	Zimbabwe- WTS $111 Malawi- WTS $23 Zimbabwe- Young people $1846 Malawi- Young people $418 Zimbabwe- Adult men $812 Malawi- Adult men $197
Choko, 2019	Empirical	Malawi	Male partners of antenatal clinic attendees	Self-test (+/− financial incentives, phone call reminders)	Facility-based testing	1 year	Health system	Cost per person treated	$35–60 (depending on financial incentive)
D’Elbée, 2020	Empirical	Lesotho	General population	HIVST added to the existing community-based HIV testing services, included a scenario with and without individual testing booths.	Facility-based testing	2 years	Health system	Cost per person tested	HIVST: $13 HIVST using individual booths: $12
								Cost per person diagnosed	HIVST: $1,033 HIVST using individual booths: $672
D’Elbée, 2021	Combined: Empiric & Modeling (for scale up costs)	Côte d’Ivoire, Senegal, Mali	Key high-risk populations (including female sex workers (FSW), men who have sex with men (MSM), and people who use drugs (PWUD))	HIVST	None	17 years	Health system	Cost per person tested	Côte d’Ivoire: $13 per WTS, $15 per MSM and $16 PWUD Senegal: $18 per WTS, $29 per MSM, $155 per PWUD Mali: $18 per WTS, $31 per MSM
D’Elbée, 2021	Combined: Empirical & Modeling (for scale up costs)	Malawi, Zambia, Zimbabwe, South Africa, Lesotho	General population (15–59 years of age)	Community-based HIVST distribution	Facility-based testing	36 months	Health system	Cost per person tested	Total sample: $14 Malawi: $10 Zambia: $13 Zimbabwe: $13 South Africa: $14 Lesotho: $13
Dovel, 2020	Empirical	Malawi	Adolescents and adults, aged ≥15 years of age	Clinic-based self-test conducted at followed by clinic-based confirmatory test, counseling and linkage to treatment	Facility-based testing (including an enhanced services arm)	4 months	Health system	Cost per person diagnosed	$237
								Cost per person treated	$363
George, 2018	Empirical	Kenya	Truckers and female sex workers who were irregular HIV testers	SMS promoted HIVST	SMS promoted facility-based testing	1 year	Health system	Cost per person tested	$10–11
Indravudh, 2021	Empirical	Malawi	Adolescents and adults (≥15 years of age)	Community based HIV self-test distribution with support for linkage to care and initiation of ART therapy	Facility-based testing	5 months	Health system	Cost per person diagnosed	$220–557
Jamieson, 2021	Modeling	South Africa	Key populations (including sex workers, taxi drivers, males attending a circumcision clinic, partners of PLHIV)	Six HIVST kit distribution modalities: Community fixed-point. Taxi ranks. Workplaces. Partner of PHC ART patients Partners of pregnant women Primary PHC	Facility-based testing	20 years	Health system	Cost per year of life saved (YLS)	$202–4,325
								Cost per HIV infection averted	$435–15,264
Maheswaran, 2016	Empirical	Malawi	General public, >18 years of age	Home-based HIVST	Facility-based testing	2 years	Health system	Cost per person diagnosed	$20
								ICER per person treated	$38–48
Maheswaran, 2018	Modelingg	Malawi	Individuals in communities with high HIV prevalence	Facility-based HIV testing and counselling plus HIVST	Facility-based testing	20 years	Health system + Patient-incurred	ICER per QALY	$52
Mangenah, 2019	Empirical	Malawi, Zambia, Zimbabwe	General public, (>16 years)	HIV self-tests performed at home, with kit use demonstration. Linkage to care was supported by a community volunteer	Facility-based testing	1 year	Health system	Cost per test	$5.91 (Malawi) $21.05 (Zambia) $7.91 (Zimbabwe)
Matsimela, 2021	Empirical	South Africa	Key populations (including sex workers, taxi drivers, males attending a circumcision clinic, partners of PLHIV)	Multiple HIVST kit distribution modalities: Horizontal PHC (ANC) Horizontal PHC (index) Vertical PHC Fixed point Mobile integration Transport hub Flexible community Key population Sex worker network Workplace (third party) Workplace (direct)	None	1 year	Health system	Cost per positive screen	$29–397
								Cost per person diagnosed	$61–1,277
								Cost per person treated	$116–5,387
Nichols, 2020	Empirical	Malawi	Adolescents and adults (>15 years)	Facility-based HIVST (with option to perform at home, if preferred)	Facility-based testing (including an enhanced services arm)	5 months	Health system	Cost per person tested	$6
								Cost per person diagnosed	$156–298
								Cost per person treated	$363
Nichols, 2022	Modeling	Malawi	Men (15–64 years) Women (15–24 years)	HIVST provided in a health care facility along with conventional FBT	FBT alone	1 year	Health system	Cost per test	1.09–4.70
								ICER per diagnosis	23–87,186
Okoboi, 2021	Empirical	Uganda	Men who have sex with men (>18 years of age)	Peer-distributed HIVST and peer-provided pre- and post-test HIV counseling	Facility-based testing	3 months	Health system	Cost per person tested	$24
								Cost per person diagnosed	$476
								Cost per averted infection	$9,161
Sande, 2021	Empirical	Malawi, South Africa, Zambia, Zimbabwe	Partners of antenatal clinic attendees or PLHIV and clients attending facility outpatient services	Both blood and oral based HIVST	None	2 years	Health system	Cost per test	$3–26 (Zambia) $5–18 (Malawi) $4–130 (South Africa)
Shahmanesh, 2021	Empirical	South Africa	Women (18–30 years)	Peer-distributed HIVST kits by both direct distribution and through financially incentivized peer networks.	Facility-based testing	7 months	Health system	Cost per person treated	$742 (peer distributed) $1,141 (financial incentive)
Traore, 2022	Empirical	Cote D’Ivoire, Mali, Senegal	Key populations (with higher underlying HIV risk)	HIVST provided in a health care facility	None	18–21 months	Health system	Cost per test	$7–8
								Cost per diagnosis	$72–705
Zachary, 2012	Modeling	Zambia	General public, >18 years of age	Clinic-based HIVST followed by confirmatory testing and counseling.	None	4 months	Health system	Cost per test	$3.28–8.17
Zishiri, 2023	Empirical	South Africa	Partners of antenatal clinic attendees Partners of PLHIV	HIVST provided in antenatal clinics and in clinics caring for PLHIV for the partners of clinic attendees Education provided to all clinic attendees regarding how to undergo HIVST	None	1 year	Health system	Cost per test	$6.69–15.76

ANC, antenatal clinic; PrEP, pre-exposure prophylaxis; HIVST, human immunodeficiency virus self-testing; SMS, short message service; DALY, disability adjusted life year; ICER, incremental cost effectiveness ratio; FBT, facility based testing; WTS, women having transactional sex; MSM, men who have sex with men; PWUD, people who use drugs; QALY, quality adjusted life year; PHC, primary health clinic.

**Table 2:** Cost-effectiveness estimates per outcome measure.

Outcome	USD, 2020
Cost/test (19–32)	1.09–155
Cost/positive screen (33)	29–397
Cost/diagnosis (21, 22, 25, 26, 32–36)	20–1,277
Cost/person treated (15, 21, 33, 34, 37)	35–5,387
Cost/HIV infection averted (26)	$9,161
ICER/test (25)	5
ICER/diagnosis (25, 30)	23–87,186
ICER/person treated (36)	38–48
ICER/DALY averted (14, 38)	Cost saving-1846
ICER/QALY (39)	52
ICER/YLS (40)	202–4,325
ICER/HIV infection averted (40)	455–15,264

HIV, human immunodeficiency virus; ICER, incremental cost-effectiveness ratio; DALY, disability-adjusted life year; QALY, quality-adjusted life year; YLS, year of life saved.

### 3.2. Quality assessment

Table 3 represents a summary of the included studies quality assessment based on the CHEER checklist (41). Nineteen of the included studies included were assessed as high quality. The remaining studies included were assessed as average quality. Further review found that most criteria were adequately reported. The criteria that were underreported included approach to engagement with patients and others affected by the study, effect of engagement with patients and others affected by the study and analytic methods used to model outcomes.

**Table 3:** Quality Assessment as per CHEERs 2022 Checklist (41).

Study	Q1	Q2	Q3	Q4	Q5	Q6	Q7	Q8	Q9	Q10	Q11	Q12	Q13	Q14	Q15	Q16	Q17	Q18	Q19	Q20	Q21	Q22	Q23	Q24	Q25	Q26	Q27	Q28	Score
Indravudh et al., 2021	Yes	Yes	Yes	Yes	Yes	Yes	Yes	Yes	Yes	Yes	Yes	Yes	Yes	Yes	Yes	No	No	Yes	No	Yes	Yes	Yes	Yes	Yes	Yes	Yes	Yes	Yes	89%
Traore et al., 2022	Yes	Yes	Yes	Yes	Yes	Yes	No	Yes	Yes	Yes	Yes	Yes	Yes	Yes	No	Yes	Yes	Yes	Yes	Yes	No	Yes	Yes	Yes	No	Yes	Yes	Yes	85%
Zishiri et al., 2023	Yes	Yes	Yes	Yes	Yes	Yes	Yes	Yes	Yes	Yes	Yes	Yes	Yes	Yes	Yes	Yes	Yes	Yes	Yes	Yes	No	Yes	Yes	Yes	No	Yes	Yes	Yes	92%
Borgstede et al., 2023	Yes	Yes	Yes	Yes	Yes	Yes	Yes	Yes	Yes	No	Yes	Yes	Yes	Yes	Yes	Yes	Yes	Yes	Yes	Yes	No	Yes	Yes	Yes	No	Yes	Yes	Yes	89%
Nichols et al., 2022	Yes	Yes	Yes	Yes	Yes	Yes	Yes	No	Yes	No	Yes	Yes	Yes	Yes	Yes	Yes	Yes	No	Yes	Yes	Yes	Yes	Yes	Yes	No	Yes	Yes	Yes	85%
Jamieson et al., 2021	Yes	Yes	Yes	Yes	No	Yes	Yes	Yes	Yes	Yes	Yes	Yes	Yes	Yes	Yes	Yes	Yes	No	No	Yes	Yes	Yes	Yes	Yes	Yes	Yes	Yes	Yes	89%
Zachary et al., 2012	No	Yes	Yes	No	Yes	Yes	Yes	No	No	No	Yes	No	Yes	No	No	No	No	No	No	No	No	Yes	Yes	No	No	Yes	Yes	Yes	43%
Shahmen et al., 2021	No	Yes	Yes	Yes	Yes	Yes	Yes	No	No	No	Yes	Yes	Yes	Yes	No	No	No	No	No	No	Yes	No	Yes	No	No	Yes	Yes	Yes	54%
Sande et al., 2021	Yes	Yes	Yes	Yes	Yes	Yes	Yes	Yes	Yes	Yes	Yes	Yes	Yes	Yes	Yes	Yes	Yes	Yes	Yes	Yes	No	Yes	Yes	Yes	No	Yes	Yes	Yes	93%
Okoboi et al., 2021	Yes	Yes	Yes	Yes	Yes	Yes	Yes	Yes	Yes	No	Yes	Yes	Yes	Yes	Yes	Yes	No	Yes	Yes	Yes	No	Yes	Yes	Yes	No	Yes	Yes	Yes	86%
Nichols et al., 2020	Yes	Yes	Yes	Yes	Yes	Yes	Yes	Yes	Yes	No	Yes	Yes	Yes	Yes	Yes	Yes	Yes	Yes	Yes	Yes	No	Yes	Yes	Yes	No	Yes	Yes	Yes	89%
Matsimela et al., 2021	Yes	Yes	Yes	Yes	Yes	Yes	Yes	Yes	Yes	Yes	Yes	No	Yes	Yes	Yes	Yes	Yes	Yes	No	Yes	No	Yes	Yes	Yes	No	Yes	Yes	Yes	86%
Mangenah et al., 2019	Yes	Yes	Yes	Yes	Yes	Yes	Yes	No	Yes	Yes	Yes	No	Yes	Yes	Yes	Yes	No	No	No	Yes	No	Yes	Yes	Yes	No	Yes	Yes	Yes	75%
Maheswaran et al., 2018	Yes	Yes	Yes	Yes	Yes	Yes	Yes	Yes	Yes	Yes	Yes	Yes	Yes	Yes	Yes	Yes	Yes	Yes	No	Yes	No	Yes	Yes	Yes	Yes	Yes	Yes	Yes	93%
Maheswaran et al., 2016	Yes	Yes	Yes	Yes	Yes	Yes	Yes	Yes	Yes	No	Yes	No	Yes	Yes	Yes	Yes	Yes	No	No	Yes	No	Yes	Yes	Yes	Yes	Yes	Yes	Yes	82%
George et al., 2021	Yes	Yes	Yes	Yes	Yes	Yes	Yes	Yes	Yes	Yes	Yes	Yes	Yes	Yes	Yes	No	No	Yes	Yes	Yes	Yes	Yes	Yes	Yes	No	Yes	Yes	Yes	89%
Dovel et al., 2020	No	Yes	Yes	Yes	Yes	Yes	Yes	Yes	No	No	Yes	Yes	Yes	Yes	Yes	No	No	No	No	No	Yes	Yes	Yes	No	No	Yes	Yes	Yes	64%
D’Elbee et al., 2021	Yes	Yes	Yes	Yes	Yes	Yes	Yes	Yes	Yes	Yes	Yes	Yes	Yes	Yes	Yes	Yes	Yes	Yes	No	Yes	No	Yes	Yes	No	No	Yes	Yes	Yes	86%
D’Elbee et al., 2020	Yes	Yes	Yes	Yes	Yes	Yes	Yes	Yes	Yes	Yes	Yes	Yes	Yes	Yes	Yes	No	No	No	Yes	Yes	No	Yes	Yes	Yes	Yes	Yes	Yes	Yes	86%
DaCruz et al., 2021	No	Yes	Yes	Yes	Yes	Yes	Yes	Yes	Yes	No	Yes	Yes	Yes	Yes	Yes	No	No	No	No	No	No	Yes	Yes	Yes	Yes	Yes	Yes	Yes	71%
Choko et al., 2019	Yes	Yes	Yes	Yes	Yes	Yes	Yes	Yes	Yes	Yes	Yes	Yes	Yes	Yes	Yes	No	No	Yes	Yes	Yes	No	Yes	Yes	No	Yes	Yes	Yes	Yes	86%
Cambiano et al., 2019	Yes	Yes	Yes	Yes	Yes	Yes	Yes	Yes	Yes	Yes	Yes	Yes	Yes	Yes	Yes	Yes	Yes	No	Yes	Yes	No	Yes	Yes	Yes	No	Yes	Yes	Yes	89%
Cambiano et al., 2015	Yes	Yes	Yes	Yes	Yes	Yes	Yes	Yes	Yes	Yes	Yes	Yes	Yes	Yes	Yes	Yes	Yes	Yes	Yes	No	Yes	Yes	Yes	Yes	No	Yes	Yes	Yes	93%
Bultery et al., 2020	Yes	Yes	Yes	Yes	Yes	Yes	Yes	Yes	Yes	Yes	No	No	Yes	Yes	Yes	Yes	Yes	Yes	No	No	No	Yes	Yes	Yes	No	Yes	Yes	Yes	79%
Amstutz et al., 2021	Yes	Yes	Yes	Yes	Yes	Yes	Yes	Yes	Yes	Yes	No	Yes	Yes	Yes	Yes	No	No	No	No	Yes	No	Yes	Yes	No	No	Yes	Yes	Yes	71%
D’Elbee et al., 2021	Yes	Yes	Yes	Yes	Yes	Yes	Yes	Yes	Yes	Yes	Yes	No	Yes	Yes	Yes	Yes	Yes	Yes	No	Yes	No	Yes	Yes	No	No	Yes	Yes	Yes	82%
Completed criteria	82%	100%	100%	95%	95%	100%	100%	86%	86%	68%	91%	73%	100%	95%	91%	59%	50%	55%	36%	73%	27%	95%	100%	68%	32%	100%	100%	100%	

(1) Title: Identify the study as an economic evaluation and specify the interventions being compared. (2) Abstract: Provide a structured summary that highlights context, key methods, results and alternative analyses. (3) Background and objectives: Give the context for the study, the study question and its practical relevance for decision making in policy or practice. (4) Methods: Indicate whether a health economic analysis plan was developed and where available. (5) Methods: Describe characteristics of the study population (such as age range, demographics, socioeconomic, or clinical characteristics). (6) Methods: Provide relevant contextual information that may influence findings. (7) Methods: Describe the interventions or strategies being compared and why chosen. (8) Methods: State the perspective(s) adopted by the study and why chosen. (9) Methods: State the time horizon for the study and why appropriate. (10) Methods: Report the discount rate(s) and reason chosen. (11) Methods Describe what outcomes were used as the measure(s) of benefit(s) and harm(s). (12) Methods: Describe how outcomes used to capture benefit(s) and harm(s) were measured. (13) Methods: Describe the population and methods used to measure and value outcomes. (14) Methods: Describe how costs were valued. (15) Methods: Report the dates of the estimated resource quantities and unit costs, plus the currency and year of conversion. (16) Methods: If modelling is used, describe in detail and why used. Report if the model is publicly available and where it can be accessed. (17) Methods: Describe any methods for analysing or statistically transforming data, any extrapolation methods, and approaches for validating any model used. (18) Methods: Describe any methods used for estimating how the results of the study vary for sub-groups. (19) Methods: Describe how impacts are distributed across different individuals or adjustments made to reflect priority populations. (20) Methods: Describe methods to characterize any sources of uncertainty in the analysis. (21) Methods: Describe any approaches to engage patients or service recipients, the general public, communities, or stakeholders (e.g., clinicians or payers) in the design of the study. (22) Results: Report all analytic inputs (e.g., values, ranges, references) including uncertainty or distributional assumptions. (23) Results: Report the mean values for the main categories of costs and outcomes of interest and summarise them in the most appropriate overall measure. (24) Results: Describe how uncertainty about analytic judgments, inputs, or projections affect findings. Report the effect of choice of discount rate and time horizon, if applicable. (25) Results: Report on any difference patient/service recipient, general public, community, or stakeholder involvement made to the approach or findings of the study. (26) Discussion: Report key findings, limitations, ethical or equity considerations not captured, and how these could impact patients, policy, or practice. (27) Other: Describe how the study was funded and any role of the funder in the identification, design, conduct, and reporting of the analysis. (28) Other: Report authors conflicts of interest according to journal or International Committee of Medical Journal Editors requirements.

### 3.3. Empirical studies

The empirical studies included HIVST interventions conducted among the general population (*n* = 14) and high-risk subgroups (*n* = 3). Most studies reported direct costs from a health care provider perspective (*n* = 15) but two studies did include patient incurred costs. HIVST was implemented in a diverse manner in both clinic and home-based settings, with a variety of different programmatic approaches to support testing and follow up. HIVST was associated with an average cost per person tested that ranged from $1.09–156. The cost of HIVST per individual diagnosed with HIV ranged from $20–1,277. There were consistently reported drivers of cost-effectiveness among studies including underlying HIV test-positivity, cost of the HIV self-test and intervention and rates of follow up and therapy initiation. Cost estimates for the same HIVST intervention varied between countries.

Results are sub-stratified by distribution strategy including clinic-based distribution where HIV self-tests were distributed in a health-care setting, door-to-door distribution where volunteers distributed HIV self-tests uniformly throughout a community or neighborhood, digital-based distribution and peer-based distribution where individuals were incentivized to distribute HIV self-tests to within their own networks.

#### 3.3.1. Clinic-based distribution

There were nine studies that implemented HIVST through a previously established health care center.

Bulterys, Choko, and Zishiri examined the cost of providing HIV self-test kits to pregnant women attending antenatal care with the aim to increase testing of their male partners (15, 19, 20) while Borgstede evaluated the cost of mailing home-based HIVST to antenatal clinic attendees. Zishiri et al. reported cost per test of approximately $7–16. Bulterys et al. found that HIVST costed $5–18 per female tested (regardless of HIV status), $19–23 per HIV-positive male and $16–20 per HIV-negative male. Choko found that costs per individual diagnosed with HIV were slightly more expensive (ranged between $35–60) because they provided a financial incentive for distribution. Choko varied the method of post-test counseling and found that the less expensive testing strategies provided post-test counseling in a group format compared to individualized post-test counseling. Borgstede et al. reported a cost of $41–49 per couple, but were able to provide home-based testing (by mailing out self-tests) along with home-based counseling and follow up.

Dovel and Nichols both published economic results from a trial that randomized outpatient clinic attendees to facility-based HIVST compared to conventional FBT (standard versus optimized) (21, 34). The facility-based HIVST provided a group demonstration and distribution of the usage of the Oraquick HIV self-test and private spaces for interpretation and counselling. The cost per person tested was higher in the HIVST group ($6.28) compared to the standard provider-initiated group ($2.44). The incremental cost-effectiveness ratio (ICER) for HIVST compared to the standard FBT was $3.21 per person tested. Cost for HIVST per new diagnosis and ART initiation were $237 and $363, respectively.

D’Elbée, Traore, and Sande assessed the costs of adding HIVST to existing community-based HIV testing services (22, 23). Sande found that cost per person tested ranged from $3–130 depending on the country of implementation. D’Elbee found that when only FBT was available, the cost was $32.2 per person tested. When both FBT and HIVST were available, the cost of FBT was $25.0 per person tested and the cost of HIVST was $15.40 per person tested. The cost per case of HIV diagnosed with HIVST was $1,033 per person diagnosed. Traore focused on key populations with an increased underlying risk of HIV and reported per test of $7–8 with cost per diagnosis of $72–705.

#### 3.3.2. Digital-based distribution

We found one study that used digital-based strategies to distribute HIV self-tests. George et al. costed HIVST using text message reminders among conventionally challenging to reach populations including male truckers and female sex workers (FSWs) in Kenya (42). HIVST, whether performed in a clinic or at home, was promoted by a text message and post- test counseling for ART initiation was provided through a clinic. Cost per case diagnosed using HIVST was $12–14 compared to $4 for facility-based testing, with an ICER of $8–10 per person tested.

#### 3.3.3. Door-to-door distribution

Four studies assessed HIVST through door-to-door distribution. These studies targeted specific geographic areas. One study specifically evaluated the cost-effectiveness of a secondary distribution to individuals who had previously declined HIVST.

Indravudh, Maheswaran, and Mangenah all examined the costs of community-led door-to-door delivery of HIV self-tests (24, 35, 39). All three studies implemented widespread delivery of HIVST kits to houses, along with worker support for linkage to care. Maheswaran also included patient-incurred costs.

Maheswaran et al. reported that the average health-care system acquired cost per person tested associated with HIVST ($1.80) was comparable to FBT ($1.53–$2.16) but that patient-incurred costs associated with HIVST were lower than in FBT. Maheswaran found that the ICER per case treated was $38–48 per additional case treated compared to FBT. The reported costs from the initial publication were higher (ICER 187–234 per case treated), however when adjusting for inflation (from 2014 to 2020) and conversion to USD, the 2020 value of the Malawian Kwacha was decreased. For Indravudh, they found that the cost per case diagnosed was on average $220, although this included previously diagnosed individuals. The average cost per case for the identification of a new HIV case was $550 per case diagnosed (which was higher than reported by Maheswaran). Mangenah reported costing outcomes only and outcome measures were not included. Cost per kit distributed ranged from $5–21 depending on the delivery model and location.

Amstutz et al. did a study in Lesotho, among the general public, to assess the impact of secondary distribution of HIVST (oral fluid) for individuals who had been absent or previously declined door-to-door HIV testing (25). They compared this to the standard of care, which was to refer absent household members to a health care facility. The interventional approach increased the test coverage substantially and accessed previously challenging to reach populations. They found that the ICER of home based HIVST was $1.34 per eligible person tested. The cost per actual person tested was lower in the HIVST arm due to increased testing coverage, with cost savings of $7 per person tested. The cost per person diagnosed with HIV with HIVST was $923, compared to $781 with the standard of care (ICER $142 per person diagnosed).

#### 3.3.4. Peer-based distribution

Two studies evaluated the cost-effectiveness of peer-distribution of HIV self-tests. One study targeted a high-risk subpopulation (MSM) while another study implemented HIVST among the general population. Both studies offered financial incentives to distribution and found that peer-based distribution was an effective way of implementing HIVST.

Okoboi et al. compared a two distribution approaches for HIVST among men who have sex with men (MSM) of HIVST via peer networks to the traditional method of performing hotspot HIVST (26). The study revealed that peer-distributed HIVST was more expensive than the SOC with respect to cost per case tested (peer-distributed vs. SOC: $24 vs. $18), though significantly less expensive for cost per newly identified case ($476 vs. $1339) and cost per averted infection ($9,161 vs. $25736). This suggests that when HIVST is distributed via peer networks among high- risk populations, it can be more efficacious and therefore cost saving compared to the facility based HIVST.

Shahmenash et al. compared standard FBT to peer distribution of HIVST kits among the general population (18–30 years old) and prospectively collected costing data as a secondary outcome (37). They found that the ICER associated with peer distributed HIVST was $114 per person initiated on ART, which the authors concluded was cost effective. Distributing HIVST through a financially incentive strategy was associated with an ICER of $513 per person initiated on ART.

#### 3.3.5. Multiple distribution strategies

Matsimela et al. conducted a micro-costing analysis of a variety of different HIVST distribution strategies in South Africa (33). They used telephone surveys, time-and-motion and expenditure analysis to estimate the cost per person tested and person diagnosed across different strategies. They found that HIVST distribution strategies across South Africa varied significantly by volume distributed, cost per kit, underlying prevalence of HIV in population tested and rates of linkage to care. These factors drove the wide variability in cost-effectiveness estimates between distribution strategies. The cost per HIVST kit varied between $5–19 and the cost per person diagnosed with HIV ranged from $61–1,277.

### 3.4. Modeling studies

We identified eight studies that used an economic model to examine the cost effectiveness of HIVST. The outcomes reported included cost per diagnosis, cost per disability adjusted life year (DALY) averted, cost per quality adjusted life year (QALY), cost per case and costs per year life saved. Two studies used a model to assess scale up costs and provided costing data with effectiveness measures. All studies concluded that HIVST could be cost-effective depending on the self-test distribution strategy and time horizon analyzed.

#### 3.4.1. Cost utility analyses

Cambiano et al. (14) used a previously published HIV transmission model to look at the impact of HIV self-testing in Zimbabwe. The study acknowledged that there was a lack of data around infrastructure costs for HIVST, but expected that HIVST would be less expensive to implement than facility based testing due to decreased need for clinic overhead. The cost savings estimate was $900 million with 7,000 DALYs averted over 20 years. When HIVST cost was increased, implementation was less cost effective. At a threshold of 48$ per ST or lower (including distribution costs), HIVST was cost effective at a willingness to pay threshold (WTP) based on the Zimbabwean GDP.

Cambiano et al. published an updated model in 2019 to examine the implementation of HIVST in the LMIC setting (38). Unit test costs for HIV ST were based off the STAR study (43) and were higher than included in their 2015 model ($5–10 per person tested). This model focused on implementation among high-risk subgroups. HIVST was cost effective among women having transactional sex with an ICER of $111 per DALY averted. The biggest impact in terms of cumulative DALYs averted was when HIV ST was implemented among adult men, with the ICER ranging from cost saving to $240 per DALY averted.

Maheswaran et al. used a Markov model to examine the cost effectiveness of HIVST in Malawi (39). They found that HIVST combined with facility-based testing was cost effective with an average ICER of $ 38,844 per QALY compared to facility-based testing alone. They included HIV related hospitalizations and comorbidities in their costs.

#### 3.4.2. Cost effectiveness analyses

Nichols et al. used regional cost data to project the cost-effectiveness of facility-based HIVST campaigns within Malawi for men and youth. They modeled of variety of different FBT HIVST strategies and estimated a cost-effectiveness ICER of 23–87,186 per additional diagnosis. This wide range of estimates was driven by differences in FBT practices between scenarios, cost of the HIVST and duration of the intervention. Jamieson et al. (40) assessed the impact of six different HIVST kit distribution modalities by a deterministic compartmental model with a horizon of 20 years in South Africa. They found that the most impactful strategy was to distribute HIVST among the partners of PLHIV however this was the least cost-effective with a cost per life year saved (LYS) of $1,448. They found that the workplace distributive model was cost-saving ($54 million to $79 million) with moderate epidemiological impact. This study suggested that the cost effectiveness of HIVST was highly impacted by the distribution strategy and subpopulation tested.

D’Elbee analyzed the expenditure of HIVST programs across Cote D’Ivoire, Senegal and Mali (27, 28). They used modeling to estimate scale up costs of HIVST across different scenarios, using data from their expenditure analysis and time motion studies. They found that programmatic and personnel represented 47–78% of HIVST cost per person, but decreased upon scale up due to the spreading of start-up costs across higher volumes. Average costs of HIVST upon scale up were $13–155 per person depending on the subpopulation. The most expensive subpopulation to reach were people who use drugs. They also published an econometric model that used key drivers of HIVST cost-effectiveness to estimate large-scale program costs across a variety of contexts. They found that the averaged predict cost per person tested was $13 (28).

Zachary and colleagues used ZamSTAR data to model the costs of introducing OraQuick, an oral HIV self-test, FBT in Zambia (29). They assumed an underlying HIV prevalence of 15% and found that the average unit cost of the self-test per person ranged between $3.28 and $8.17 depending on the testing algorithm. This included downstream confirmatory testing but did not include treatment related costs for those who self-tested positive.

## 4. Discussion

HIVST is being increasingly used as a strategy to improve the uptake of HIV diagnosis, especially among subgroups who face barriers to accessing facility-based healthcare. We identified 25 studies that evaluated the economics of HIVST. The majority were empirical studies that included a costing component. Six studies modeled the cost-effectiveness of HIVST (14, 38, 39), while two used a model to predict scale up costs (27).

The cost per case diagnosed with HIV ranged from $20–$1,277 per case diagnosed. The range in cost estimates, likely results from the differences in the costs and implementation approaches included in each study. For example, the estimate of $20 per case represents the cost of HIVST promoted by volunteers with testing done within the home and excludes downstream diagnostic and treatment costs while the estimate of $1,277 per case represents the cost of HIVST facilitated by a mobile phone program and includes downstream diagnostic and treatment costs (33, 36).

All studies concluded that despite increased unit costs for HIVST compared to FBT, that HIVST was cost-effective in at least one scenario investigated. Four studies actually found that HIVST was actually cost-saving compared to conventional HIV testing (25, 26, 38, 40).

The testing algorithm among studies, was similar with either home or facility based HIVST followed by confirmatory testing and counseling. However, elements included in costing analyses varied widely between studies. Programmatic costs were frequently not reported, yet when included typically represented a large portion of the total cost (27). Reported outcomes included cost per test, cost per case diagnosed, cost per case treated, ICER per case diagnosed/treated, cost per infection averted, cost per years of life saved, cost per QALY and cost per DALY averted. The heterogeneity of the costs and outcomes made it impossible to conduct a meta-analysis, which is why we have summarized the results in narrative format.

Particularly among the modeling studies, we were unable to broadly group results as the eight studies reported six different outcomes (cost per diagnosis, cost per DALY averted, cost per YLS, cost per case, cost per QALY, scale-up costs). Only three (3/25) studies included utility outcome measures, such as QALYs and DALYs, which allows for standardization and comparison of outcomes across studies. The costs included in the HIVST intervention were also variable. Ideally studies reporting costs should aim to include overhead programmatic, costs associated with implementation and personnel costs, to avoid underestimating the total expenses associated with HIVST. The heterogeneity of our results, suggest the need for standardized conduct of economic evaluations to improve the generalizability across studies. Standardized reporting guidelines exist but barriers exist to achieving these standards, including study design, financial resources, and investigator time.

We have stratified our studies by distribution strategy and outcome measure. The most common distribution strategy was clinic-based, which used a pre-existing health care facility to implement HIVST. Door-to-door distribution was also popular and involved the use of community volunteers to uniformly disperse HIVST. Compared to community-based distribution, door-to-door methods had higher uptake of HIVST but lower linkage to care. Two studies focused on HIVST distribution through peer-based networks (26, 37). They found that peer distribution of HIVST and educational materials was a cost-effective method compared to clinic-based distribution of HIVST. Peer based distribution, particularly among high-risk subgroups, may help to overcome the barriers that certain populations may face in accessing conventional based HIV diagnosis.

Most included studies (24/25) used community-based methods to support HIVST through distribution of tests and linkage of those who self-tested positive to further care. There was a relative lack of cost-effectiveness literature around digital based HIVST, with only one study evaluating digital-based programs in a LMIC context. Recent reviews have suggested that digital based HIVST is increasing on a global level and may help to support linkage to care and ART initiation after self-testing (12). The WHO and the G20 India presidency recently launched the “Global Initiative on Digital Health” which will focus on digital-based health-care services on a global level (44). Given the increasing importance of digital-based health services future studies that describe the cost effective of HIVST paired with digital based support are of utmost relevance.

### 4.1. Strengths and limitations

Our study summarizes a large body of literature on HIVST and is the first systematic review to summarize the economic literature behind HIVST, however there are several important limitations. The field of HIVST is rapidly evolving and it is likely that some of the earlier publications do not reflect the current cost effectiveness realities. For example, the cost of HIV self-test kits was subsidized in 2017 by the Bill and Melinda Gates Foundation (<$2) which will change the cost effectiveness profile among many countries (45). The cost of HIVST diagnostics and ART vary between countries and are impacted by national negotiations and drug procurement strategies. To mitigate this, we have limited our search strategy to the past 10 years. Additionally, there is likely an element of publication bias as efficacious and cost-effective interventions are more likely to be published. This would skew our results to overestimate the cost effectiveness of HIVST.

We limited our search strategy to low- and middle-income countries because of significant differences in HIV-related diagnosis and treatment costs between locations. By excluding high-income countries, we were able to consolidate cost-estimates per outcome measure. Our systematic review includes 25 publications from 10 different countries. Interestingly, all publications were based out of the continent of Africa. Recently there have been multiple cost-effectiveness analyses of HIVST published from higher income settings, such as the United States, China and Brazil (46–49). These studies report higher costs associated with HIVST, but have also concluded that HIVST can be cost-effective. These studies have focused on key populations, with higher underlying risk of HIV test-positivity.

Economic studies are context specific and generalizing across locations should be done with caution. The wide range of estimates reported across studies highlights the multiple factors that can drive cost-effectiveness. In studies that reported on the same HIVST across multiple settings, estimates varied significantly (23, 24, 27, 28). Many studies identified underlying prevalence of undiagnosed HIV as an important driver of cost effectiveness (14, 15, 38, 39). This is highly dependent on the subpopulation, which is why HIVST tends to be more cost effectiveness among groups with higher underlying risk of contracting HIV. Other drivers of cost-effectiveness were the cost of the self-test, uptake and rates of follow up to confirmatory testing and care and the cost of ART.

In the future, it would valuable to understand how drivers of cost-effectiveness interact with each other and the location of self-testing. It would be helpful to identify thresholds of cost-effectiveness, for example the cost per self-test or for ART treatment required to keep HIVST cost-effective. More long-term cost-effectiveness data is required, particularly if HIVST campaigns are to be maintained over a period of years. There is some modeling data that suggests that longer duration of HIVST is increasingly cost-effective (30), but most empirical studies have focused on short-term interventions. Finally, further economic data on digital (versus community-based) means of HIVST would be important as these increasingly being used on a global scale.

## 5. Conclusion

HIVST is a novel approach that has been shown to improve the uptake and acceptability of HIV testing in high risk and general populations. It is especially promising among high -risk subgroups that face barriers in accessing facility-based care, due to perceived stigma and discrimination, but these populations a high underlying prevalence of undiagnosed HIV. This systematic review represents the first summary of the economic evidence for HIVST. Our findings suggest that HIVST can be cost effective in an LMIC context, particularly among key populations.

## Data availability statement

The original contributions presented in the study are included in the article/supplementary material, further inquiries can be directed to the corresponding author.

## Author contributions

BE, AbK, and AnK performed the systematic review and data extraction. BE and AA performed the quality assessment. BE wrote the manuscript. BE, Ank, AbK, O’DS, SC, MS, AA, NP, and AZ edited the manuscript. All authors have seen and approved the submitted manuscript and involved in the project synthesis and developing the general methodology.
